# Gut Microbiota-Derived Short Chain Fatty Acids Are Associated with Clinical Pregnancy Outcome in Women Undergoing IVF/ICSI-ET: A Retrospective Study

**DOI:** 10.3390/nu15092143

**Published:** 2023-04-29

**Authors:** Xinrui Yao, Sitong Dong, Wenzheng Guan, Lingjie Fu, Gaoyu Li, Zhen Wang, Jiao Jiao, Xiuxia Wang

**Affiliations:** 1 Center of Reproductive Medicine, Shengjing Hospital of China Medical University, 39 Huaxiang Road, Shenyang 110004, China; 2 Shenyang Reproductive Health Clinical Medicine Research Center, Shenyang 110004, China; 3 Department of Research and Development, Germountx Company, Beijing 102200, China; 4 The Research Center for Medical Genomics, School of Life Sciences, China Medical University, No.77 Puhe Road, Shenyang North New Area, Shenyang 110122, China; 5 Key Laboratory of Reproductive and Genetic Medicine, China Medical University, National Health Commission, Shenyang 110004, China

**Keywords:** short chain fatty acids, IVF/ICSI-ET, clinical pregnancy outcome

## Abstract

Gut microbiota and its metabolites are related to the female reproductive system. Animal experiments have demonstrated the relationship between gut microbiota-derived short chain fatty acids (SCFAs) and embryo quality. However, few studies have linked SCFAs to clinical pregnancy outcomes in humans. This retrospective cross-sectional study recruited 147 patients undergoing in vitro fertilization or intracytoplasmic sperm injection and embryo transfer (IVF/ICSI-ET) (70 with no pregnancies and 77 with clinical pregnancies). The association between SCFAs levels and clinical pregnancy outcomes was evaluated using univariate and multivariate logistic regression analyses. The association between SCFAs and metabolic parameters was analyzed using a linear regression model. Receiver operating characteristic (ROC) curve analysis was used for assessing the efficiency of SCFAs to evaluate the clinical pregnancy outcomes. Fecal propionate levels were significantly higher in the no pregnancy group than in the clinical pregnancy group (*p* < 0.01). Fecal acetate and butyrate levels were not significantly different between females with and without clinical pregnancies (*p* > 0.05). There were positive relationships between fecal propionate levels and fasting serum insulin (FSI) (r = 0.245, *p* = 0.003), Homeostatic Model Assessment for Insulin Resistance (HOMA-IR) (r = 0.276, *p* = 0.001), and triglycerides (TG) (r = 0.254, *p* = 0.002). Multivariate analyses determined that fecal propionate (OR, 1.103; 95% CI, 1.045–1.164; *p* < 0.001) was an independent risk factor for no pregnancies. The area under the ROC curve (AUC) of fecal propionate was 0.702 (*p* < 0.001), with a sensitivity of 57.1% and a specificity of 79.2%. High fecal propionate concentration has a negative association on clinical pregnancy outcomes and is positively correlated with FSI, TG, and HOMA-IR.

## 1. Introduction

Infertility is defined as a female of childbearing age with a normal and unprotected sexual life who has not been pregnant for 1 year after copulation, and 8–12% of couples of reproductive age are affected globally [1,2]. In recent years, assisted reproductive technologies (ARTs), such as artificial insemination (AIH), in vitro fertilization or intracytoplasmic sperm injection (IVF/ICSI), have been used to treat infertility. Although ART has made impressive advancements, the clinical pregnancy outcomes remain relatively unsatisfactory, especially for in vitro fertilization or intracytoplasmic sperm injection-embryo transfer (IVF/ICSI-ET) [3]. Hence, improving the clinical pregnancy rate of patients undergoing IVF/ICSI-ET has become a major clinical challenge [4].

The gut microbiota is a varied community of bacteria that dwells in the digestive tracts of humans and animals and plays an important part in modulating systemic metabolic functions of the host, including female reproductive function [5,6]. Previous studies have demonstrated a link between gut microbiota abnormalities and female reproductive disorders, i.e., polycystic ovary syndrome (PCOS), endometriosis, and ovarian dysfunction, and conditions such as insulin resistance, obesity, and hyperglycemia, which affect reproductive function [7,8,9,10]. In addition, gamete generation, conceptus implantation, placentation, abortion, and metabolic reprogramming throughout crucial life cycle stages are aspects of embryonic development that might be influenced by the gut microbiota [6]. However, the underlying mechanisms involved remain unclear.

The generation of metabolites by gut microbiota affects the physiology of the host, with the main products being short-chain fatty acids (SCFAs) [11], including butyrate, propionate, and acetate. Primary carbon transfer from food through the gut microbiota to the host is represented by SCFAs, and there is growing evidence that SCFAs regulate glucose and lipid metabolism and immune function [12]. Animal experiments have shown that SCFAs play crucial roles in embryo implantation and growth. For example, Ye et al. [13] showed that sodium butyrate supplementation markedly increased live litter size, embryo implantation, and survival in rats by increasing progesterone production. G protein-coupled receptors 41 (GPR41) and 43 (GPR43) that control the growing neurological system, pancreatic beta cells, and the enteroendocrine system, are influenced by propionate during mouse development [14]. This suggests that SCFAs may be key mediators of the effect of the gut microbiota on female reproductive function and embryonic development, which are essential for clinical pregnancy. However, few studies have linked SCFAs with clinical pregnancy outcomes in women undergoing IVF/ICSI-ET. Therefore, we conducted a hospital-based retrospective case-control study to evaluate the relationship between SCFAs and clinical pregnancy outcomes for the first time.

## 2. Materials and Methods

### 2.1. Study Design and Study Population

**Figure 1:**
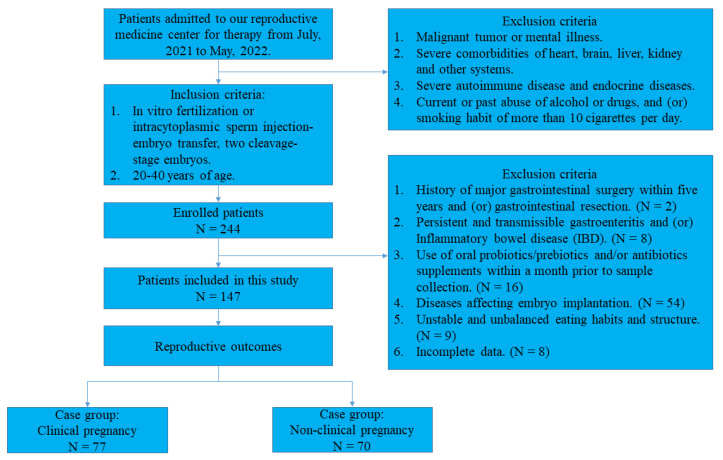
The inclusion and exclusion criteria of the study population.

This retrospective case-control study was conducted at a single center. The research cohort included 244 females experiencing infertility who underwent IVF/ICSI-ET at the Reproductive Medicine Center of Shengjing Hospital of China Medical University between July 2021 and May 2022. The study design was approved by the Ethical Review Board of Shengjing Hospital, China Medical University (approval no. 2021PS016F), in accordance with the Declaration of Helsinki. All participants provided written informed consent prior to participation. General information and clinical parameters of the patients were obtained from an electronic medical record database. As factors such as intestinal diseases, previous antibiotic use, and changing diet are known to temporarily affect the intestinal microbial population, participants provided relevant information by means of a questionnaire at the time of inclusion to correct for possible confounders. The criteria for inclusion were as follows: (1) women between the ages of 20 and 40 who are infertile; and (2) women undergoing IVF/ICSI-ET using two cleavage-stage embryos. The exclusion criteria were as follows: (1) malignant tumor or mental illness; (2) severe comorbidities of heart, brain, liver, kidney, and other systems; (3) severe autoimmune disease and endocrine diseases; (4) a history of major gastrointestinal surgery within 5 years and (or) gastrointestinal resection; (5) persistent and transmissible gastroenteritis and (or) inflammatory bowel disease; (6) the use of oral probiotics/prebiotics and/or antibiotic supplements within a month prior to sample collection; (7) diseases affecting embryo implantation such as hydrosalpinx, uterine fibroids, adenomyosis, endometrial polyp, uterine mal-formations; (8) unstable and unbalanced eating habits and structure; and (9) incomplete data. Clinical pregnancy is defined as an intrauterine pregnancy sac and fetal heartbeat observed using a transvaginal ultrasound 35 d after embryo transfer. Ultimately, 147 participants (70 with no pregnancies and 77 with clinical pregnancies) were enrolled in the study. A general description of the inclusion and exclusion criteria is shown in Figure 1.

### 2.2. Blood Sample Collection and Measurement

On the third and fifth days of the initial spontaneous menstrual period, blood samples were collected in the morning after a night of fasting. Using the UniCelDxI 800 Automated Immunoassay Platform (Beckman Coulter, Brea, CA, USA), and following the manufacturer’s guidelines, the following hormones were evaluated: estradiol (E2), progesterone (P), prolactin (PRL), total testosterone (TT), follicle-stimulating hormone (FSH), luteinizing hormone (LH), and anti-Müllerian hormone (AMH). The clinical parameters assessed via an enzyme-linked immunosorbent assay on an ARCHITECT ci16200 Automatic Biochemical Analyzer (Abbott Laboratories, Chicago, IL, USA) included sex hormone-binding globulin (SHBG), fasting serum insulin (FSI), fasting plasma glucose (FPG), total cholesterol (CHOL), triglycerides (TG), high-density lipoprotein cholesterol (HDL-C), low-density lipoprotein cholesterol (LDL-C), total bilirubin (TBIL), and total bile acid (TBA). The homeostatic model assessment for insulin resistance (HOMA-IR) was performed using the formula (FSI, U/mL × FPG, mmol/L)/22.5.

### 2.3. Fecal Sample Collection and Determination of SCFA Concentrations

Throughout the study, stool specimens were collected 3 d after the acquisition of blood samples, with participants fasting for a minimum of 8 h. Using the HALO-F100 fecal processing device (Hailu Biotechnology Co., Ltd., Suzhou, China), a specified amount of fecal matter (0.800 ± 0.010 g) was treated to generate a 10% fecal suspension. Subsequently, a 2 mL aliquot was prepared and subjected to centrifugation at 10,000 rpm for 5 min at 4 °C in a sterile, new centrifuge tube (Thermo Fisher Scientific Co., Ltd., Waltham, MA, USA). The resulting supernatant was transferred to a separate centrifuge tube and thoroughly mixed. Thereafter, 500 μL of the liquid was pipetted into a 1.5 mL centrifuge tube, combined with 100 μL of a SCFA pretreatment solution, and stored at −30 °C for 24 h. After thawing the sample, the supernatant was isolated, filtered, and centrifuged once more at 10,000 rpm at 4 °C for 3 min. The analytical process was completed by transferring 100 μL of the sample into a gas chromatography sample vial (Fuli 9720, Taizhou, China). The gas chromatography parameters were established as follows: chromatographic column (Agilent FFAP 30 m × 0.25 mm × 0.25 μm); column temperature (20 °C/min to 180 °C for 1 min, 50 °C/min to 220 °C for 1 min); injector (ultra-pure nitrogen, 250 °C, 1.0 μL, split ratio 5:1; a flow rate of 2.5 mL/min for 6.5 min, and 2.8 mL/min for 2 min); detector (FID, temperature: 250 °C, hydrogen: 30 mL/min, oxygen: 300 mL/min).

### 2.4. Statistical Analyses

A Kolmogorov-Smirnov assessment was conducted to evaluate the normality of continuous variable distributions. For normally distributed continuous variables; the mean and standard deviation were used; whereas the median and interquartile range represented continuous variables with non-normal distributions. The Mann-Whitney U test and Student’s *t*-test were used to analyze normal and non-normal continuous variables; respectively. Categorical data were examined using Fisher’s exact test and the chi-square test. Pearson correlation coefficients were used to determine the relationship between two quantitative variables. To analyze the impact of significant factors on clinical pregnancy outcomes, univariate and multivariate logistic regression analyses were performed, calculating the odds ratio (OR) and confidence interval (CI) for the presence of a non-pregnancy. Receiver operating characteristic (ROC) curve analysis was used for assessing the efficiency of pertinent factors to evaluate the clinical pregnancy outcomes. In addition, the sensitivity, specificity, and area under the ROC curve (AUC) with 95% CIs were calculated. Statistical analyses were conducted using the Statistical Package for Social Sciences; Version 25 (IBM Corp.; Armonk; New York, NY, USA). Results were considered significant when *p* was less than 0.05 in all two-sided tests.

## 3. Results

### 3.1. Comparison of Baseline Characteristics between the Two Study Populations

The baseline characteristics of the clinical and no pregnancy groups are shown in Table 1. Those who had a clinical pregnancy outcome had more 2PN oocytes than patients who did not (*p* = 0.033). Table 2 shows that patients with no pregnancy had significantly higher FSI (*p* = 0.001), HOMA-IR (*p* = 0.002), and TG (*p* = 0.016) values than patients with clinical pregnancy.

### 3.2. Fecal Propionate Levels Correlated with FSI, HOMA-IR, and TG

**Figure 2:**
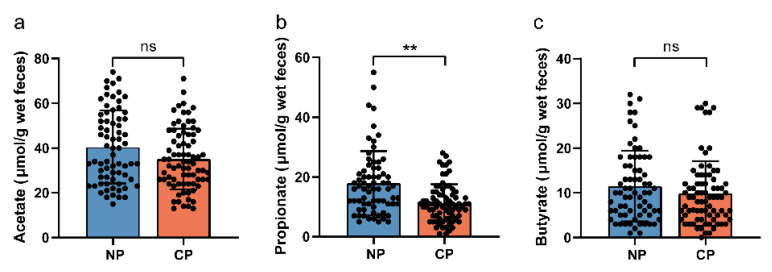
Differences in fecal acetate, propionate, and butyrate levels in patients with no pregnancy and clinical pregnancy. (**a**) Comparison of fecal acetate levels between clinical and no pregnant groups. (**b**) Comparison of fecal propionate levels between clinical and no pregnant groups. (**c**) Comparison of fecal butyrate levels between clinical and no pregnant groups. NP, no pregnancy; CP, clinical pregnancy. ns, no significant. ** *p* < 0.01.

**Figure 3:**
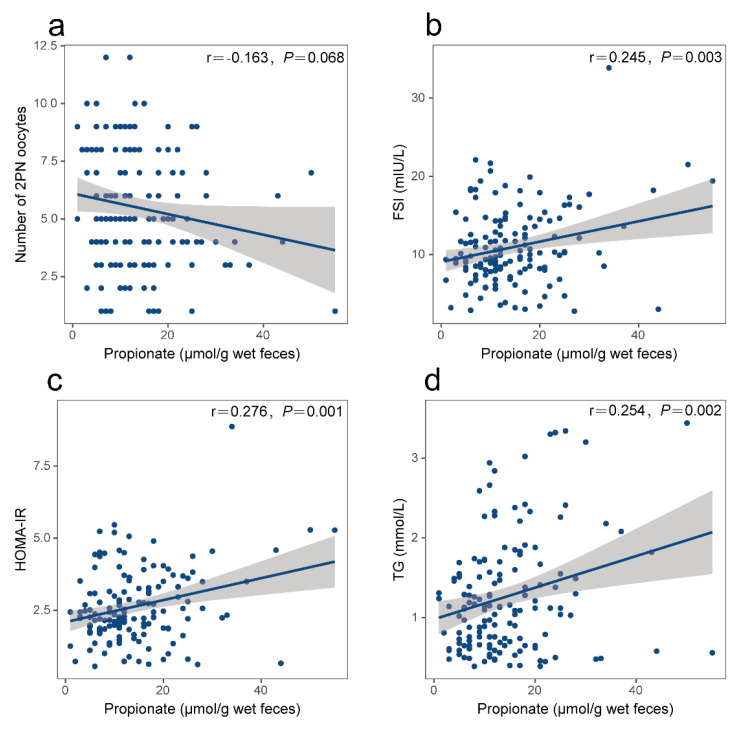
Correlation between fecal propionate and the number of 2PN oocytes, FSI, HOMA-IR, and TG in enrolled patients. (**a**) Linear correlation between fecal propionate and the number of 2PN oocytes. (**b**) Linear correlation between fecal propionate and FSI. (**c**) Linear correlation between fecal propionate and HOMA-IR. (**d**) Linear correlation between fecal propionate and TG. TG, triglycerides; HOMA-IR, homeostatic model assessment for insulin resistance; FSI, fasting serum insulin.

In this study, we quantified SCFAs, namely acetate, propionate, and butyrate, from the fecal specimens of our subjects, and the results are depicted in Figure 2. A notable elevation in fecal propionate concentrations was observed in the no pregnancy cohort compared to the clinical pregnancy cohort (*p* < 0.01); however, no significant differences were found in the fecal acetate and butyrate concentrations between two groups. We investigated the association between fecal propionate and the significant parameters we mentioned above, including the number of 2PN oocytes, FSI, HOMA-IR, and TG levels, using Pearson’s linear regression analysis. A linear correlation assessment revealed positive correlations between fecal propionate levels and FSI (r = 0.245, *p* = 0.003), HOMA-IR (r = 0.276, *p* = 0.001), and TG (r = 0.254, *p* = 0.002) (Figure 3b–d). No significant correlation was observed between fecal propionate levels and the number of 2PN oocytes. The fecal acetate, propionate, and butyrate levels in each participant are shown in Supplementary Table S1. We also analyzed the correlation between fecal propionate and FSI, HOMA-IR, and TG in the clinical pregnancy and no pregnancy groups separately (Supplementary Figure S1).

### 3.3. Fecal Propionate Is a Risk Factor for No Pregnancy

**Figure 4:**
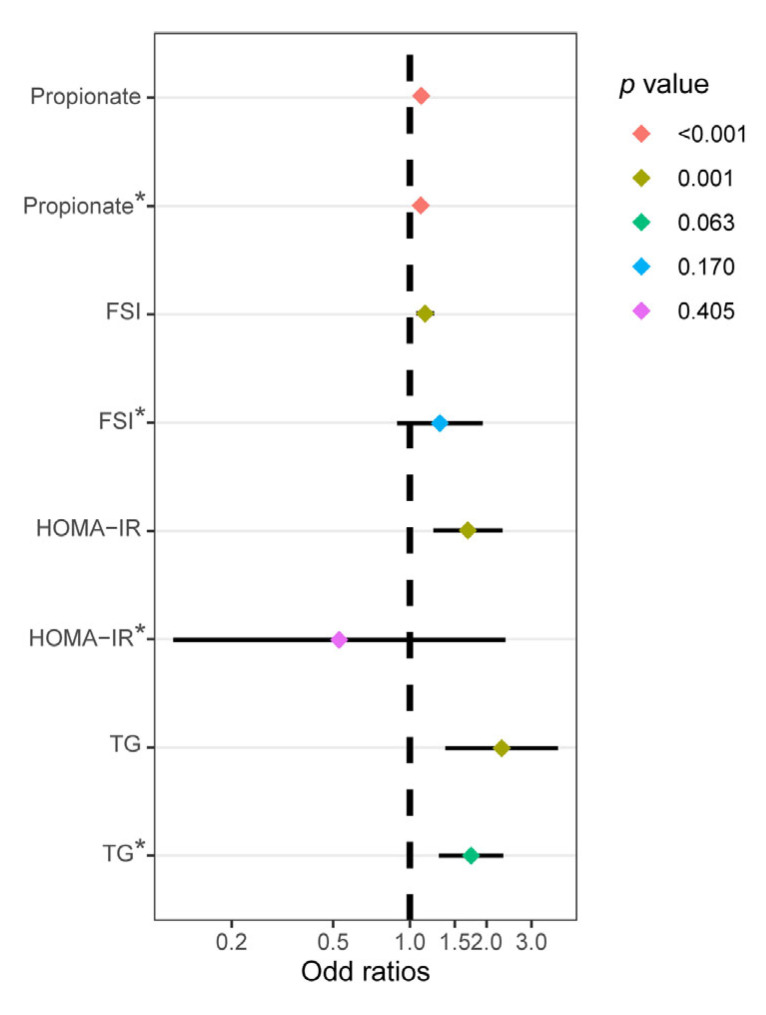
Univariate and multivariate logistic regression analyses of the variables influencing clinical pregnancy outcomes. FSI, fasting serum insulin; HOMA-IR, homeostatic model assessment for insulin resistance; TG, triglycerides. * The *p* value was adjusted for the other three factors.

Using logistic regression analysis, we assessed the association between propionate levels and the prevalence of no pregnancy in patients (Figure 4 and Table 3). Regarding clinical pregnancy outcome, univariate analyses identified fecal propionate (OR, 1.108; 95% CI, 1.054–1.165; *p* < 0.001), FSI (OR, 1.147; 95% CI, 1.059–1.244; *p* = 0.001), HOMA-IR (OR, 1.688; 95% CI, 1.236–2.307; *p* = 0.001), and TG (OR, 2.291; 95% CI, 1.378–3.809; *p* = 0.001) as risk factors affecting clinical pregnancy outcomes. Multivariable analyses determined that after adjusting for covariates (FSI, HOMA-IR, and TG), fecal propionate (OR, 1.103; 95% CI, 1.045–1.164; *p* < 0.001) was an independent risk factor for no pregnancy.

**Figure 5:**
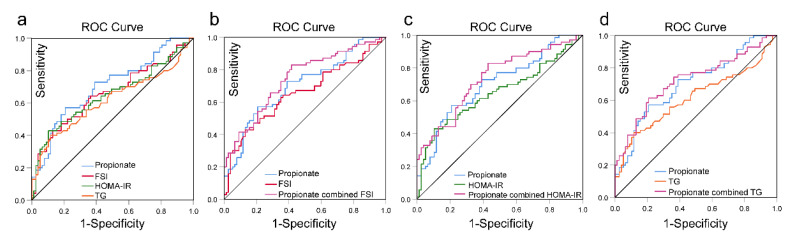
The performance of fecal propionate, FSI, HOMA-IR, and TG in evaluating pregnancy outcomes. (**a**) ROC analysis of propionate, FSI, HOMA-IR, and TG to determine effectiveness in pregnancy outcomes. (**b**) ROC analysis of propionate, FSI, and a combination of propionate and FSI to determine effectiveness in pregnancy outcomes. (**c**) ROC analysis of propionate, HOMA-IR, and a combination of propionate and HOMA-IR to determine effectiveness in pregnancy outcomes. (**d**) ROC analysis of propionate, TG, and a combination of propionate and TG to determine effectiveness in pregnancy outcomes. ROC, receiver operating characteristic; AUC, area under the curve; TG, triglycerides; FSI, fasting serum insulin; HOMA-IR, homeostatic model assessment for insulin resistance.

We evaluated the performance of fecal propionate in distinguishing patients with clinical pregnancy and no pregnancy using ROC curves. As demonstrated in Table 4 and Figure 5a, the AUC of fecal propionate (>14.97 mol/g wet feces) was 0.702 (*p* < 0.001) with a sensitivity of 57.1% and a specificity of 79.2%. Regarding the other significant markers, FSI (>13.14 mIU/L) had an AUC of 0.655 (*p* = 0.001), with a sensitivity of 42.9% and a specificity of 87.0%. The AUC of HOMA-IR (>3.29) was 0.648 (*p* = 0.002), with a sensitivity of 42.9% and a specificity of 89.6% for distinguishing no pregnancy. TG (>3.29 mmol/L) had an AUC of 0.615 (*p* = 0.016), a sensitivity of 42.9%, and a specificity of 89.6%. 

In addition, the combination of fecal propionate and FSI had an AUC of 0.743, with a sensitivity of 82.9% and a specificity of 58.4% for clinical pregnancy outcome evaluation (Table 4 and Figure 5b). The AUC of the combination of fecal propionate and HOMA-IR (AUC = 0.735) was higher than that of HOMA-IR alone (AUC = 0.648) (Figure 5c). The combination of fecal propionate and TG showed a higher AUC (0.722) and sensitivity value (61.4%) than TG alone (AUC = 0.615) (Figure 5d).

## 4. Discussion

In the present study, the fecal SCFAs levels and metabolic parameters were investigated in the patients undergoing IVF/ICSI. We found that fecal propionate levels were significantly elevated in women with no pregnancy compared to women with clinical pregnancy. Strikingly, we observed that fecal propionate might be a risk factor for clinical pregnancy outcomes, even after adjusting for the relevant covariates FSI, HOMA-IR, and TG.

With the development and deepening of gut microbiota research in the field of female reproductive function, SCFAs, as the main metabolites of gut microbiota, have begun to be widely investigated. In our present research, elevated fecal propionate was associated with clinical pregnancy failure. In a clinical study [15], compared with pregnant women with normal BMI, fecal propionate in overweight/obese pregnant women was significantly higher (*p* = 0.022). There was a significant positive correlation between evaluated fecal propionate and the plasma glucose concentration and glycated hemoglobin (HbA1) in the group of obese pregnant women. Those results suggested that propionate might play a role in the pregnancy process, but the mechanism still needs further elaboration.

Our results found that fecal propionate was positively correlated with FSI, HOMA-IR, and TG. Previous research reported that fecal propionate was a regulator for glycolipid metabolic indicators. As one of main products of the gut microbial fermentation of non-digestible ingredients such as dietary fiber and resistant starch [16], the fecal propionate levels are influenced by diet structure. Considering circulating biomarkers of cardiometabolic health such as HOMA-IR, TG and low-density lipoprotein (LDL) are also related to the diet structure [17,18,19], suggesting that fecal propionate might play the mediator role in dietary intake and in those markers of glycolipid metabolism. Previous studies have certified that elevated FSI and TG levels in early pregnancy were associated with adverse pregnancy outcomes, such as gestational diabetes mellitus and large-for-gestational-age, and can be used as reliable markers of adverse pregnancy outcomes [20,21,22]. It has been suggested that hyperglycemia affects embryonic development and increases the vulnerability of embryos to cardiac malformations [23]. According to Wang et al., the TG levels of women negatively correlated with the quantity of oocytes, cleaved embryos, normally fertilized oocytes, and high-quality embryos [24]. According to a study conducted by Song et al. [25], the clinical pregnancy rate negatively correlated with increased HOMA-IR, which is an important predictor, particularly for infertile women without PCOS during IVF. The underlying mechanism is that high insulin levels can adversely affect the preimplantation environment by downregulating the expression of IGF-binding protein-1 and glycodelin, which play important roles in the interaction between the embryo and endometrium [26,27].

Combining our results with the above discussion, the mechanism underlying the effect of fecal propionate on clinical pregnancy may be explained. As the main fermentation metabolite of gut microbiota, fecal propionate levels may represent those in the systemic circulation and portal [28]. Compared to other SCFAs, propionate demonstrated the greatest difference between obese and lean participants, with propionate levels significantly higher in obese participants [29]. Meanwhile, propionate was found to be the most symbolic SCFA of persons who are prone to obesity by the variable importance in projection (VIP) score [30]. As reported by Emanuel et al. [31], propionate may affect lipoprotein lipase inhibitors, such as angiopoietin-like 4 (ANGPTL4), increasing free fatty acid uptake. Regulated by a GPR43-related mechanism, propionate may increase PPARγ-mediated adipogenesis. Propionate also decreased intracellular lipolysis by reducing HSL phosphorylation. Collectively, these effects would lead to elevated TG levels that are regulated by propionate. Although some studies have shown that intestinal microbiota-derived SCFAs have beneficial glucose metabolic effects [32,33], according to a study by Vishal et al. [34], increased propionate produced by gut microbiota and transported to the liver through the portal vein controls metabolic disorders in Toll-like receptor 5-deficient mice. Propionate may cause glycogenolysis, insulin resistance, and hyperglycemia by increasing plasma levels of glucagon, insulin counter-regulatory hormones, and fatty acid-binding protein 4 (FABP4) in both mice and humans [35]. Therefore, various pathways may be responsible for the effects of propionate on glucose metabolism, and fecal propionate may alter clinical pregnancy outcomes by affecting lipid and glucose metabolism. Supplementary Figure S2 illustrates this process.

However, it must be acknowledged that our research has several limitations. The primary disadvantage of our investigation was the small number of samples included. To assess the varied effects of propionate on clinical pregnancy more accurately and to aid in the development of more unbiased findings, more research involving larger human populations is required. Although we screened the participants based on their eating habits and structure, diet was a crucial factor in gut microbiota-derived SCFA production. Follow-up studies should aim to complete a more detailed assessment of the participants’ diet, such as grams of meat, bean products, vegetables, and fruits consumed, as well as the total calorie intake per day. In addition, the present research only included data on SCFAs from infertile women undergoing IVF/ICSI-ET, and excluded those healthy women spontaneously conceived without any fertility treatment; hence, we were unable to characterize the SCFAs of these populations. Previous studies have shown that the gut microbiota of infertile women undergoing ARTs is altered in abundance and quantity compared to the healthy controls [36,37], providing the theoretical support for our future research on gut microbiota-derived SCFAs and female infertility.

In summary, our current study demonstrated that a higher concentration of fecal propionate has a negative effect on clinical pregnancy outcomes and is positively correlated with FSI, TG, and HOMA-IR. These preliminary results provide new insights and directions for future studies of dietary nutrition and gut microbiota in female infertility.

## Figures and Tables

**Table 1:** Baseline characteristics of the no pregnancy and clinical pregnancy groups.

	No Pregnancy (*n* = 70)	Clinical Pregnancy (*n* = 77)	*p* Value
Age (years)	33.57 ± 3.96	33.90 ± 2.97	0.573 ^a^
BMI (kg/m^2^)	24.34 ± 3.34	23.38 ± 2.75	0.059 ^a^
Infertility duration (years)	3.00 (2.00–5.00)	3.00 (2.00–6.00)	0.905 ^b^
Education, N (%)			0.104 ^c^
Middle school and below	14 (20.00)	7 (9.09)	
High school	3 (4.29)	10 (12.99)	
College	46 (65.71)	53 (68.83)	
Master’s and above	7 (10.00)	7 (9.09)	
Dietary habits (*n*, %)			0.316 ^c^
Vegetarian diet	2 (2.86)	7 (9.10)	
Meat-based diet	5 (7.14)	6 (12.99)	
Meat and vegetarian diet	63 (90.00)	64 (77.91)	
Ovulation regimen (*n*, %)			0.152 ^c^
Long	0 (0)	1 (1.30)	
Super-long	9 (12.86)	3 (3.90)	
Antagonist	49 (70.00)	60 (77.92)	
PPOS	12 (17.14)	13 (16.88)	
Fertilization (*n*, %)			0.596 ^c^
IVF	31 (44.29)	31 (40.26)	
ICSI	35 (50.00)	38 (49.35)	
IVF + ICSI	4 (5.71)	8 (10.39)	
Previous pregnancies (*n*, %)	37 (52.86)	31 (40.26)	0.126 ^d^
Endometrial thickness (mm)	9.38 ± 2.20	9.89 ± 2.13	0.150 ^a^
Number of oocytes retrieved	7.00 (5.00–9.00)	8.00 (6.00–10.00)	0.074 ^b^
Number of 2PN oocytes	5.00 (3.00–7.00)	6.00 (4.00–8.00)	0.033 ^b^
Number of MII oocytes	5.00 (3.00–7.00)	6.00 (4.00–8.00)	0.095 ^b^
Number of suitable embryos	4.00 (2.00–5.00)	4.00 (2.00–7.00)	0.093 ^b^

BMI, body mass index; PPOS, progestin-primed ovarian stimulation; IVF, in vitro fertilization; ICSI, intracytoplasmic sperm injection. Data that are normally distributed are shown as the mean standard deviation (SD), whereas data that are not normally distributed are shown as the median (interquartile range); ratio (the number of occurrences/the total number of events) is the presentation method for proportional data. ^a^ for the Student’s *t*-test. ^b^ for the Mann-Whitney U test. ^c^ for the Fisher’s exact test. ^d^ for the Chi-square test.

**Table 2:** Basic clinical parameters of the no pregnancy and clinical pregnancy groups.

	No Pregnancy (*n* = 70)	Clinical Pregnancy (*n* = 77)	*p* Value
E2 (pg/mL)	31.65 (24.07–44.37)	34.62 (26.04–52.97)	0.165 ^a^
P (ng/mL)	0.46 (0.30–0.71)	0.54 (0.36–0.66)	0.563 ^a^
PRL (ng/mL)	13.83 (10.23–15.62)	12.01 (9.43–16.79)	0.337 ^a^
SHBG (nmol/L)	30.65 (20.18–56.50)	38.80 (25.95–49.75)	0.416 ^a^
TT (ng/mL)	0.46 (0.35–0.54)	0.44 (0.35–0.52)	0.695 ^a^
FSH (mIU/mL)	7.39 (6.17–8.56)	7.30 (6.03–8.21)	0.646 ^a^
LH (mIU/mL)	4.17 (2.81–5.83)	3.67 (2.43–6.26)	0.786 ^a^
AMH (ng/mL)	3.25 (1.63–5.04)	3.92 (2.40–5.99)	0.054 ^a^
FSI (mIU/L)	11.65 (8.50–16.40)	9.35 (7.54–11.61)	0.001 ^a^
FPG (mmol/L)	5.39 ± 0.57	5.35 ± 0.48	0.665 ^b^
HOMA-IR	2.76 (1.92–4.00)	2.22 (1.79–2.75)	0.002 ^a^
CHOL (mmol/L)	4.77 (4.31–5.15)	4.66 (4.28–5.23)	0.958 ^a^
TG (mmol/L)	1.28 (0.67–2.11)	0.97 (0.66–1.35)	0.016 ^a^
HDL-C (mmol/L)	1.38 ± 0.29	1.41 ± 0.30	0.638 ^b^
LDL-C (mmol/L)	2.93 (2.46–3.30)	2.91 (2.37–3.45)	0.998 ^a^
TBIL (μmol/L)	9.90 (8.25–12.93)	11.10 (8.90–13.05)	0.167 ^a^
DBIL (μmol/L)	2.70 (1.98–3.30)	2.70 (2.10–3.50)	0.441 ^a^
IDBIL (μmol/L)	7.40 (5.88–9.73)	8.40 (6.60–9.65)	0.192 ^a^
TBA (μmol/L)	2.11 (1.28–3.07)	1.78 (1.08–3.47)	0.687 ^a^

Estradiol (E2), progesterone (P), prolactin (PRL), sex hormone-binding globulin (SHBG), total testosterone (TT), luteinizing hormone (LH), anti-Müllerian hormone (AMH), fasting serum insulin (FSI), fasting plasma glucose (FPG), and the homeostatic model assessment for insulin resistance (HOMA-IR). TBIL, total bilirubin; DBIL, direct bilirubin; IDBIL, indirect bilirubin; HDL-C, high-density lipoprotein cholesterol; TG, triglycerides; LDL-C, low-density lipoprotein cholesterol; TBA, total bile acid. The mean and standard deviation (SD) of normally distributed data are reported, and the median value is reported for data that are not normally distributed (interquartile range). ^a^ for the Mann-Whitney U test. ^b^ for the Student’s *t*-test.

**Table 3:** Univariate and multivariate logistic regression analyses evaluating the factors affecting clinical pregnancy outcomes.

	Univariate Regression			Multivariate Regression		
	OR	95%CI	*p* Value	OR *	95%CI	*p* Value
Propionate (μmol/g wet feces)	1.108	1.054–1.165	<0.001	1.103	1.045–1.164	<0.001 ^a^
FSI (mIU/L)	1.147	1.059–1.244	0.001	1.311	0.891–1.929	0.170 ^b^
HOMA-IR	1.688	1.236–2.307	0.001	0.528	0.118–2.372	0.405 ^c^
TG (mmol/L)	2.291	1.378–3.809	0.001	1.738	0.971–3.112	0.063 ^d^

FSI, fasting serum insulin; HOMA-IR, homeostatic model assessment for insulin resistance; TG, triglycerides; OR, odds ratio; CI, confidence interval. * odds ratio after adjustment. ^a^ *p* value is adjusted for FSI, HOMA-IR, and TG. ^b^ *p* value is adjusted for fecal propionate, HOMA-IR, and TG. ^c^ *p* value is adjusted for fecal propionate, FSI, and TG. ^d^ *p* value is adjusted for fecal propionate, FSI, and HOMA-IR.

**Table 4:** The performance of fecal propionate, FSI, HOMA-IR, and TG in evaluating pregnancy outcomes.

	AUC	SE	Cut off	95% CI	Sensitivity%	Specificity%	*p* Value
Propionate	0.702	0.043	14.97	0.617–0.786	57.1	79.2	<0.001
FSI	0.655	0.046	13.14	0.565–0.745	42.9	87.0	0.001
HOMA-IR	0.648	0.047	3.29	0.556–0.739	42.9	89.6	0.002
TG	0.615	0.048	3.29	0.521–0.709	42.9	89.6	0.016
Propionate + FSI	0.743	0.041	0.36	0.663–0.822	82.9	58.4	<0.001
Propionate + HOMA-IR	0.735	0.041	0.36	0.655–0.816	82.9	57.1	<0.001
Propionate + TG	0.722	0.043	0.49	0.638–0.805	61.4	79.2	<0.001

TG, triglyceride; FSI, fasting serum insulin; HOMA-IR, homeostatic model assessment for insulin resistance; AUC, area under the curve; SE, standard error; CI, confidence interval.

## Data Availability

The data used to support the findings of this study are available from the corresponding authors upon request.

## Supplementary Materials

The following supporting information can be downloaded at: https://www.mdpi.com/article/10.3390/nu15092143/s1, Figure S1: Correlation between fecal propionate and FSI, HOMA-IR, and TG in patients with no pregnancy and clinical pregnancy. Figure S2: Propionate may influence clinical pregnancy outcomes by mediating glycolipid metabolism. Table S1: The fecal acetate, propionate, and butyrate levels in enrolled participants.
