# Supplementary figures and images for: Gut Microbiota-Derived Short Chain Fatty Acids Are Associated with Clinical Pregnancy Outcome in Women Undergoing IVF/ICSI-ET: A Retrospective Study
Source: Nutrients. 2023 Apr 29;15(9):2143. doi: 10.3390/nu15092143 (PMC10180850; doi:10.3390/nu15092143)

**a**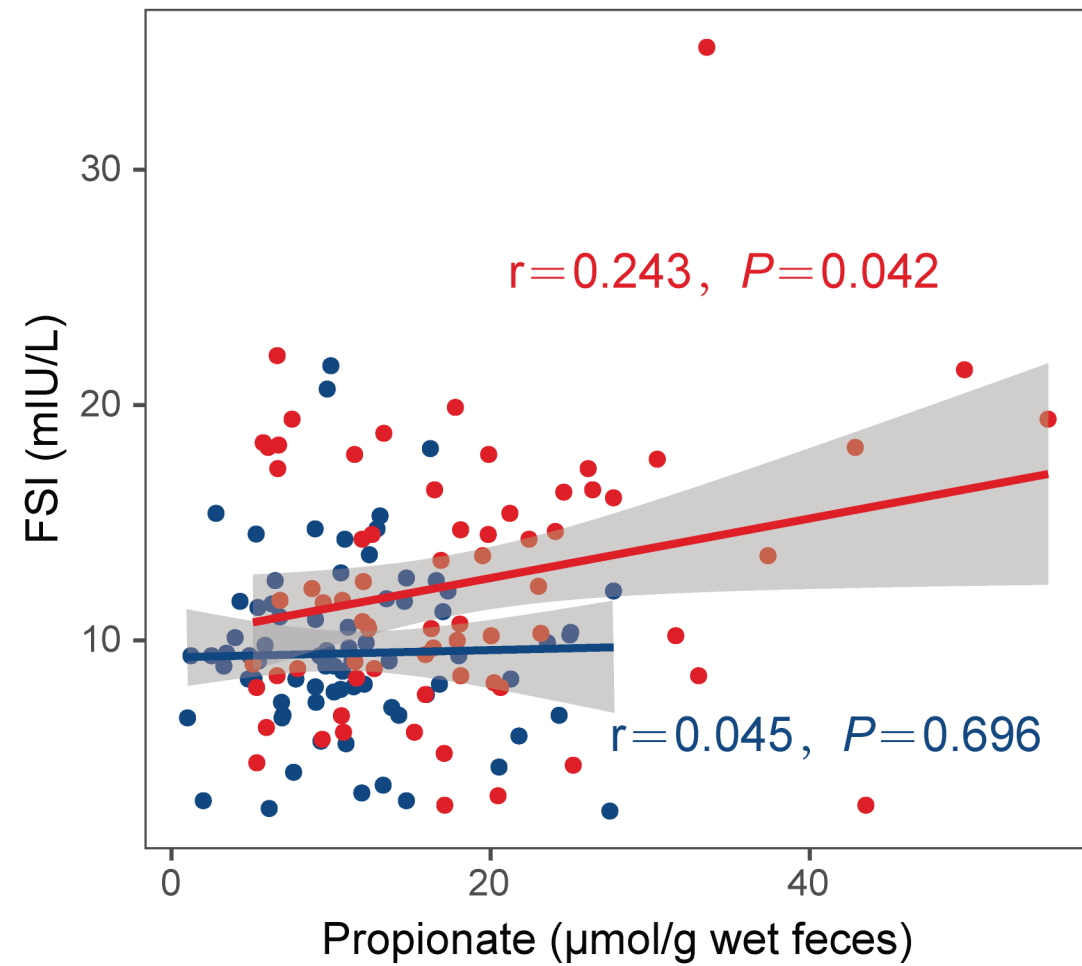**b**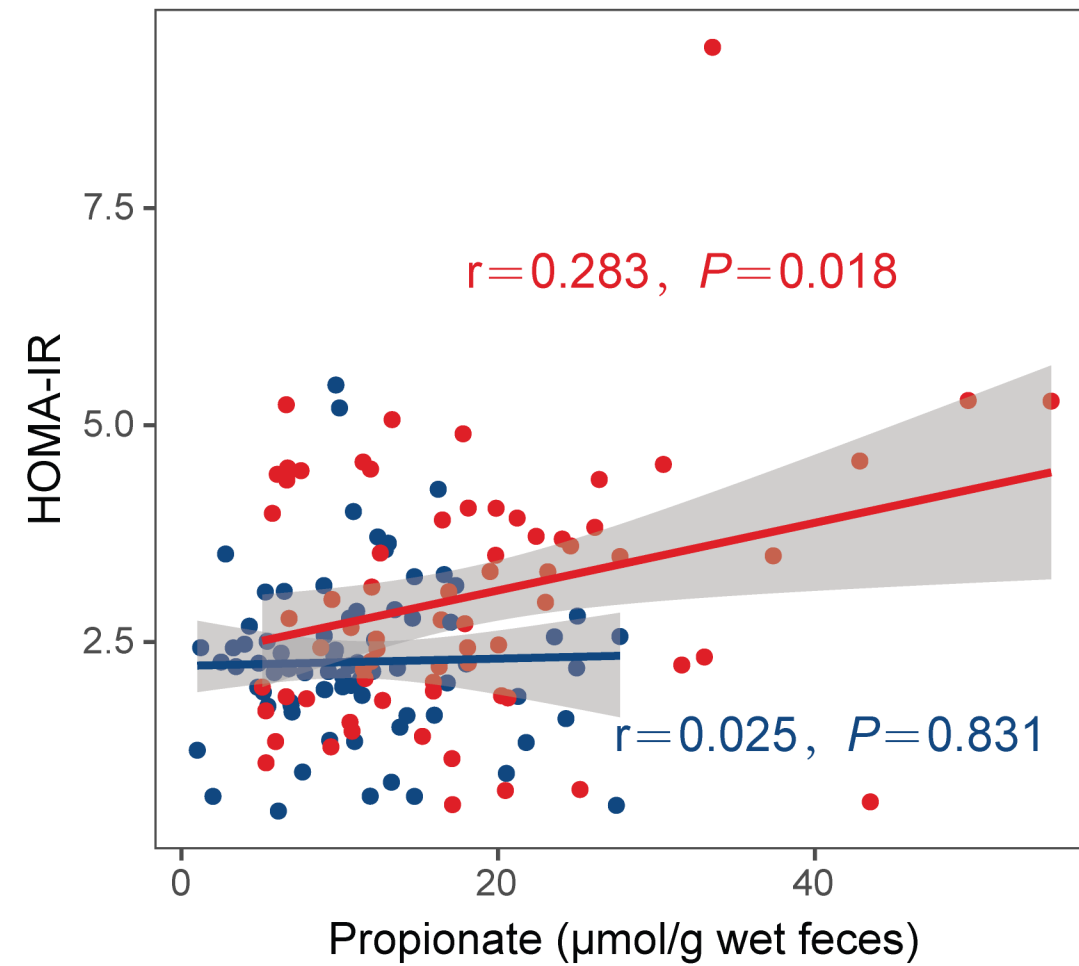**c**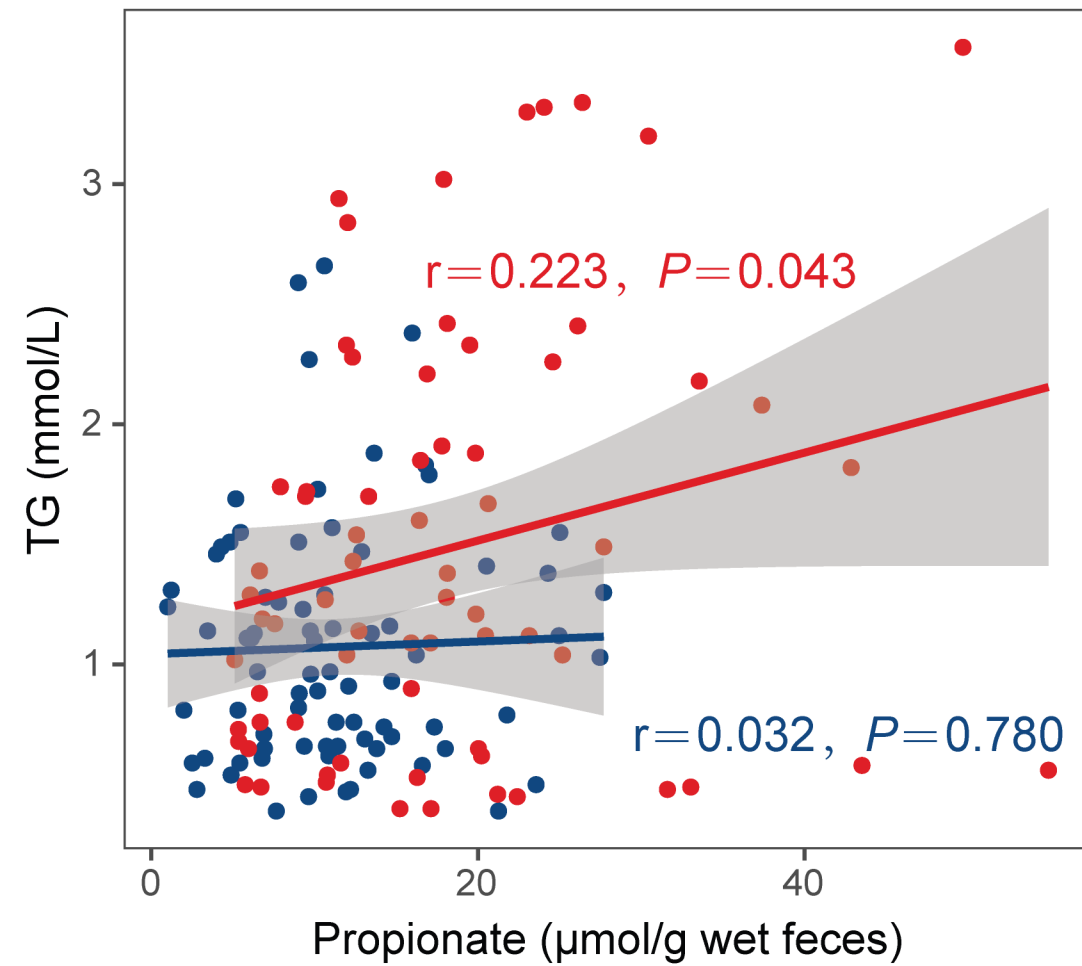

Supplement: Supplementary file 1 [file nutrients-15-02143-s001.zip › Supplementary Figrue S1.pdf]

# Clinical pregnancy failure

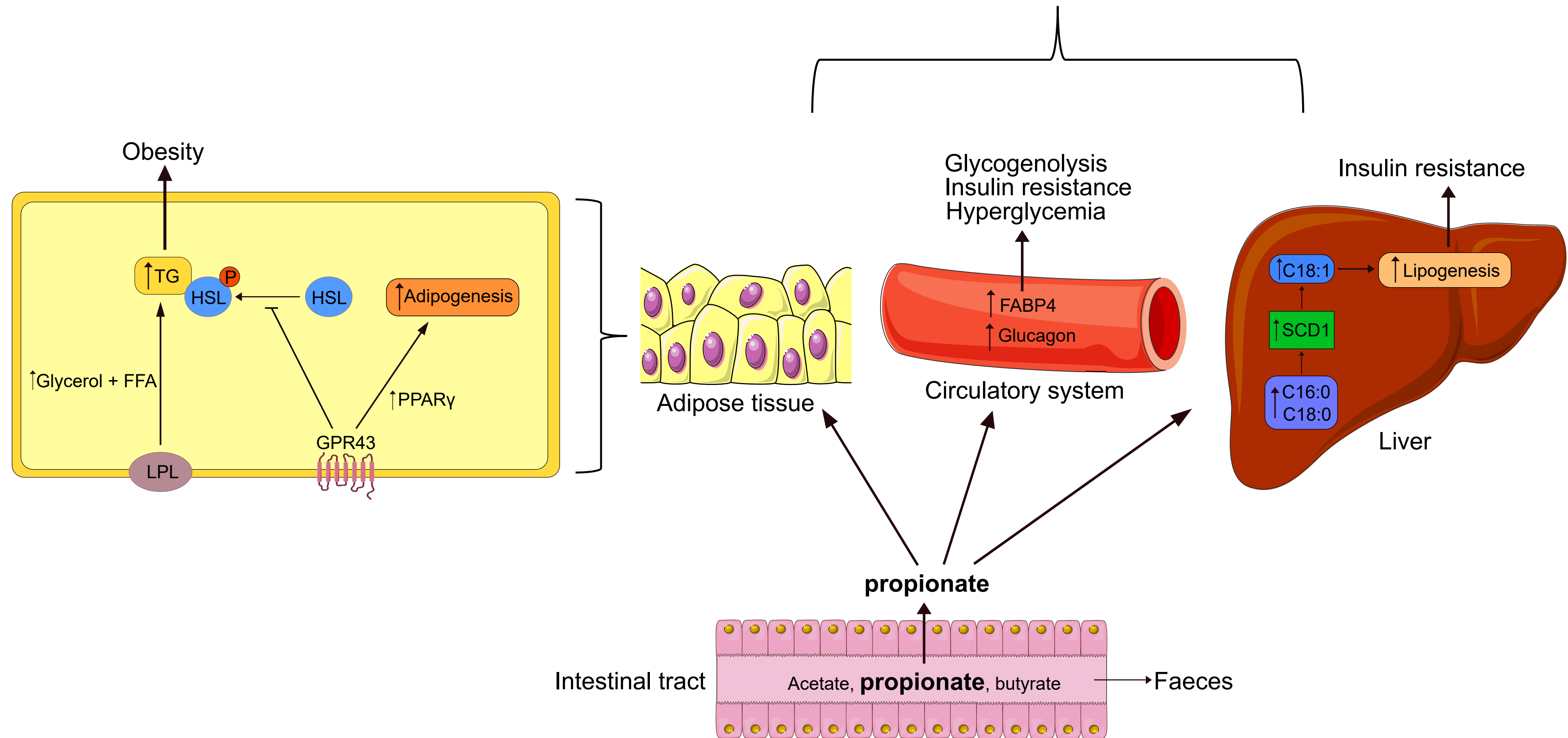

Supplement: Supplementary file 1 [file nutrients-15-02143-s001.zip › Supplementary Figrue S2.pdf]
